# Organizing the bacterial annotation space with amino acid sequence embeddings

**DOI:** 10.1186/s12859-022-04930-5

**Published:** 2022-09-23

**Authors:** Susanna R. Grigson, Jody C. McKerral, James G. Mitchell, Robert A. Edwards

**Affiliations:** Flinders Accelerator for Microbiome Exploration, College of Science and Engineering, Flinders University, Adelaide, South Australia 5042 Australia

**Keywords:** Function prediction, Machine learning, Sequence embedding, Protein ontology, Bacteria

## Abstract

**Background:**

Due to the ever-expanding gap between the number of proteins being discovered and their functional characterization, protein function inference remains a fundamental challenge in computational biology. Currently, known protein annotations are organized in human-curated ontologies, however, all possible protein functions may not be organized accurately. Meanwhile, recent advancements in natural language processing and machine learning have developed models which embed amino acid sequences as vectors in *n*-dimensional space. So far, these embeddings have primarily been used to classify protein sequences using manually constructed protein classification schemes.

**Results:**

In this work, we describe the use of amino acid sequence embeddings as a systematic framework for studying protein ontologies. Using a sequence embedding, we show that the bacterial carbohydrate metabolism class within the SEED annotation system contains 48 clusters of embedded sequences despite this class containing 29 functional labels. Furthermore, by embedding *Bacillus* amino acid sequences with unknown functions, we show that these unknown sequences form clusters that are likely to have similar biological roles.

**Conclusions:**

This study demonstrates that amino acid sequence embeddings may be a powerful tool for developing more robust ontologies for annotating protein sequence data. In addition, embeddings may be beneficial for clustering protein sequences with unknown functions and selecting optimal candidate proteins to characterize experimentally.

**Supplementary Information:**

The online version contains supplementary material available at 10.1186/s12859-022-04930-5.

## Background

Bacteria are ubiquitous across the biosphere and fulfil crucial roles in environmental, clinical, and industrial settings [1–3]. With the advent of low-cost, high throughput DNA sequencing technologies and metagenomic studies, the amino acid sequences of millions of bacterial proteins have been obtained [4, 5]. This data represents a valuable resource for expanding our knowledge of bacteria across many environments and developing new biotechnologies. Despite this, current practices for inferring the function of novel protein sequences involve costly and time-intensive in vitro and in vivo experiments [6]. As a result, an ever-increasing gap persists between the number of known bacterial protein sequences and known protein functions.

To annotate sequence data with functional labels, computational pipelines have been developed which assign functional annotations to amino acid sequences. These pipelines rely on ontologies of protein functional annotations curated into hierarchies by human experts. Commonly used ontologies include Gene Ontology [7] and the Kyoto Encyclopedia of Genes and Genomes, with the SEED annotation system [8] the preferred ontology for bacterial protein sequences. Specifically, SEED contains four hierarchical levels of functional annotations referred to as superclasses, classes, subclasses and subsystems.

Using these ontologies, annotation pipelines usually assign protein functions by comparing the homology of novel protein sequences to sequences with known functions. This approach generally relies on similarity-based algorithms such as BLAST or profile hidden Markov models [9, 10]. An estimated one-third of all known bacterial proteins have no known homologs, limiting the number of annotations that can be accurately predicted [11]. Additionally, annotations from high-throughput experiments are often strongly biased towards a limited number of functions, leaving large regions of the protein space unexplored [12].

Recent advancements in machine learning have enabled the development of sequence embeddings, which predict protein functions using algebraic representations of amino acid sequences. Rather than relying on sequence homology, these methods apply natural language processing models designed to analyze text to biological sequence data. A straightforward approach is to apply *k-*mer frequency, or *k*-mer counts, where the number of occurrences of each *k*-mer in a protein sequence is used to group sequences with similar biological properties [13, 14]. This method does not require training a machine learning model, however, it produces large, sparse matrices at a high computational cost. An alternative sequence embedding method is Protvec which converts amino acid sequences to overlapping subsequences of length *k* (*k-*mers) and applies the *word2vec* algorithm to embed amino acid sequences as vectors within a 100-dimensional space [15]. As Protvec cycles through training data, it learns biophysical and biochemical properties of amino acid sequences and generates a hyperspace based on these features. As a result, sequences with similar biological functions are proximally located within this space. This quality has been leveraged to train classifiers that produce alignment-free, protein function predictions [16, 17].

As sequence embeddings have quickly become a useful tool for a range of protein prediction tasks [18–20], ongoing research focuses on developing more advanced sequence embeddings. This frequently involves applying novel natural language processing algorithms to protein sequence data and reporting high predictive capabilities [21–24]. However, functional predictions based on sequence embeddings rely on annotation ontologies constructed using incomplete knowledge of possible protein functions. Theoretically, sequence embeddings could be used as a systematic framework for improving protein annotation ontologies by clustering and comparing amino acid sequences without relying on homology.

Here, we explore the potential use of protein sequence embeddings as a tool for evaluating and improving bacterial annotation ontologies. By embedding and subsequently clustering amino acid sequences involved in carbohydrate metabolism from the bacterial genus, *Bacillus,* we identify inconsistencies between the hierarchical organization of embedded sequences and their SEED annotations. Additionally, we demonstrate that sequence embeddings can be used to produce clusters of unknown bacterial protein sequences which likely possess similar biological functions. The concepts proposed in this study present previously unexplored uses of sequence embeddings, beneficial for generating a homology-free framework that facilitates higher accuracy annotations of bacterial proteins.

## Methods

### Data collection and filtering

*Bacillus* amino acid sequences were obtained from the *Genome Taxonomy Database* (GTDB) (release 95) [25] and the *Pathosystems Resources Integration Center* (PATRIC) [26] and annotated with SEED annotations [8] using PATRIC [26]. Sequences were filtered by removing: sequences containing an ambiguous amino acid denoted by an ‘X’, sequences shorter than 30 amino acids, and sequences longer than 1024 amino acids. These size limits were selected as sequences shorter than 30 amino acids are unlikely to form a protein domain and sequences longer than 1024 amino acids are uncommon [27–29].

### Strategy

Sequence embeddings were used to evaluate the organization of *Bacillus* sequences in the SEED carbohydrate metabolism class. A Protvec model was trained using all the filtered *Bacillus* sequences from the GTDB database which were annotated with the carbohydrate metabolism SEED class (8743 sequences). Filtered *Bacillus* sequences from PATRIC which were also annotated with the carbohydrate metabolism SEED class were embedded using this model, excluding the sequences which were also present in GTDB and used to train the model (24,836 sequences). This same set of sequences was also embedded using *k*-mer frequency and a Protvec model trained with 324,018 sequences from the Swiss-Prot database in previous work [15, 30, 31]. For all three of these embedding methods, the number of clusters present was evaluated. The hierarchical organization of the sequences in the embedding which showed the greatest clustering (the *Bacillus* Protvec model embedding) was then compared with the SEED annotations of the sequences.

Protvec was also used to embed *Bacillus* sequences with unknown functions. This was achieved using the filtered *Bacillus* sequences, previously downloaded from PATRIC which were not assigned a SEED annotation (4,155,438 sequences). These sequences were dereplicated at 70% identity using CDHIT [32] to remove redundant sequences, resulting in 824,463 distinct sequences. To reduce computational requirements, a random sample of 450,000 of these sequences was generated using *seqtk* (https://github.com/lh3/seqtk). A second random sample of 425,000 unannotated sequences was generated from this sample of 450,000 sequences using *seqtk* and used to train a Protvec model. The remaining 25,000 unannotated sequences which were not used for training were then embedded using this model.

To compare the embedded groupings of unknown sequences with known sequences, the *Bacillus* carbohydrate metabolism sequences from PATRIC which were embedded previously (24,836 sequences) were also embedded with the Protvec model trained with unannotated *Bacillus* sequences.

### Protvec models

Protvec models were trained by generating all possible 3-mers for a set of training sequences by representing each sequence as three lists of shifted, non-overlapping 3-mers. Using the Python *gensim* implementation of *word2vec* [33] (https://github.com/RaRe-Technologies/gensim), these 3-mers were trained through a skip-gram neural network with a vector size of 100 and a context size of 25 to produce a 100-dimensional vector for each 3-mer present in the training data.

The length of each 3-mer vector in the *Bacillus* carbohydrate metabolism Protvec model was calculated as the Euclidean distance of each vector from the origin. The kernel density estimate distribution of these lengths was calculated using the python *SciPy* package [34]. The model was then compared with the BLOSUM62 Matrix (BLOck Substitution) [35] by determining the count of each amino acid amongst the 100 longest 3-mer vectors and comparing these counts with the value of each amino acid on the diagonal of the BLOSUM62 matrix.

### Protvec embedding

**Fig. 1:**
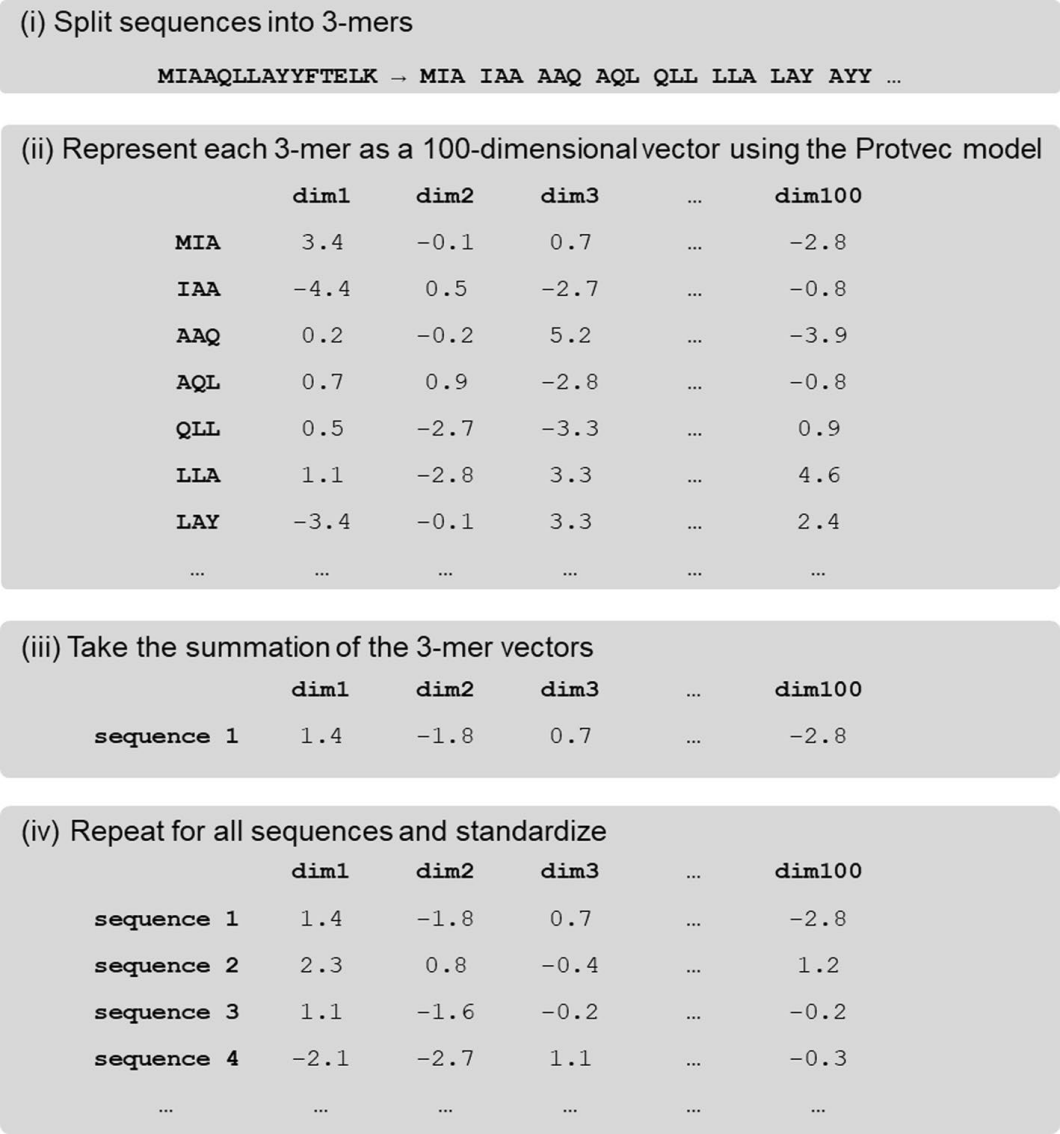
Procedure to embed amino acid sequences as vectors using Protvec models

Sequences were embedded using Protvec models by converting sequences to strings of overlapping 3-mers*,* matching each 3-mer to the corresponding vector in the Protvec model and taking the summation of these vectors. The embedded sequence vectors were standardized using Z-score normalization with the Python *scikit-learn* package [36] (Fig. 1).

Sequence embedding vectors were visualized using principal component analysis (PCA). Each sequence vector was colored by its SEED subclass annotation to visualize the grouping of sequences with similar biological roles.

### K-mer frequency embedding

Sequences were also embedded using the frequency of each *k*-mer in a sequence. As *k-*mer count matrices of amino acid sequences are sparse, sequences were converted to the murphy10 reduced amino acid alphabet [37]. This alphabet reduces the standard amino acid alphabet which contains 20 characters to only 10 characters, {A, C, G, H, P, L, S, F}, which could be used to fold all protein sequences. Using the murphy10 alphabet resulted in 10^3^ possible 3-mers, meanwhile, 20^3^ possible 3*-*mers exist for the standard amino acid alphabet.

All 10^3^ possible 3-mers were represented as a zero vector with a 1 at a unique position by labelling the rows of an identity matrix of size *n* = 10^3^ with each possible 3-mer (Additional File 1: Fig. S1). To embed sequences, the vectors in this matrix corresponding to the 3-mers in each sequence were summed and then normalized by dividing by the length of the sequence. The resulting sequence vectors were visualized using PCA with each sequence colored according to its SEED subclass annotation.

### Cluster analysis

The number of clusters present in sequence embeddings was estimated using the Calinski-Harabasz index [38]. Agglomerative clustering on the Euclidean distances between sequence vectors was applied to 5000 randomly selected sequences embedded using the *Bacillus* Protvec model, *k*-mer frequency, and the Swiss-Prot Protvec model to create dendrograms which were cut into *K* clusters for *K* = 2:150. For 500 bootstraps of each *K,* the within-cluster sum of squares (*WSS*) (1) and the between-cluster sum of squares (*BSS*) (2) were calculated,

1 $$WSS\left( {\text{K}} \right) = \sum\nolimits_{{k = 1}}^{K} {\sum\nolimits_{{i = 1}}^{{n_{k} }}} ||x_{ik} - \overline{{x_{k} }}||^{2}$$

2 $$BSS\left( {\text{K}} \right) = \sum\nolimits_{{k = 1}}^{K} {{n_{k} }} || {\overline{{x_{k} }} - \bar{x}} || ^{2}$$

where $n$ is the total number of elements, $n_{k}$ is the number of elements in the $k$ th cluster, x_*ik*_ refers to the *i* th element in the *k* th cluster, $\overline{{x_{k} }}$ is the mean of the $k$ th cluster and $\overline{{x_{ } }}$ is the sample mean. Using the $WSS$ and $BSS$ the CH index (3) was calculated for each *K.*

3 $$CH\left( {\text{K}} \right) = \frac{BSS\left( K \right)}{{WSS\left( K \right)}}\frac{{\left( {n - k} \right)}}{{\left( {k - 1} \right)}}$$

The resulting CH index was plotted for each embedding and the optimal number was clusters was determined as the value of *K* where the CH index peaks.

### Hierarchical clustering

The organization of sequences embedded with the *Bacillus* carbohydrate metabolism Protvec model was compared with their SEED annotations. To do this, 1000 *Bacillus* carbohydrate metabolism sequences which were embedded previously were randomly selected using *seqtk *(https://github.com/lh3/seqtk). A hierarchy was built using agglomerative clustering on the Euclidean distance between sequences using the R *cluster* library. This hierarchy was compared with the SEED annotations of the sequences by building a tanglegram with the R *dendextend* library [39]. This tanglegram was untangled using the *step2side* method to minimize entanglement between the constructed hierarchy and SEED annotations.

### Clustering unknowns

The embedded *Bacillus* sequences with unknown functions were clustered using *k-*means clustering and visualized using *t*-distributed Stochastic Neighborhood Embedding (*t*-SNE) [40]. *k-*means clustering was used rather than agglomerative clustering for unknown sequences as the clusters may not be functionally related. To determine the similarity of sequences within each unknown cluster, the sequence similarity of the unknown embedded sequences was evaluated using Clustal Omega [41].

## Results

### Analysis of the protein space

**Fig. 2:**
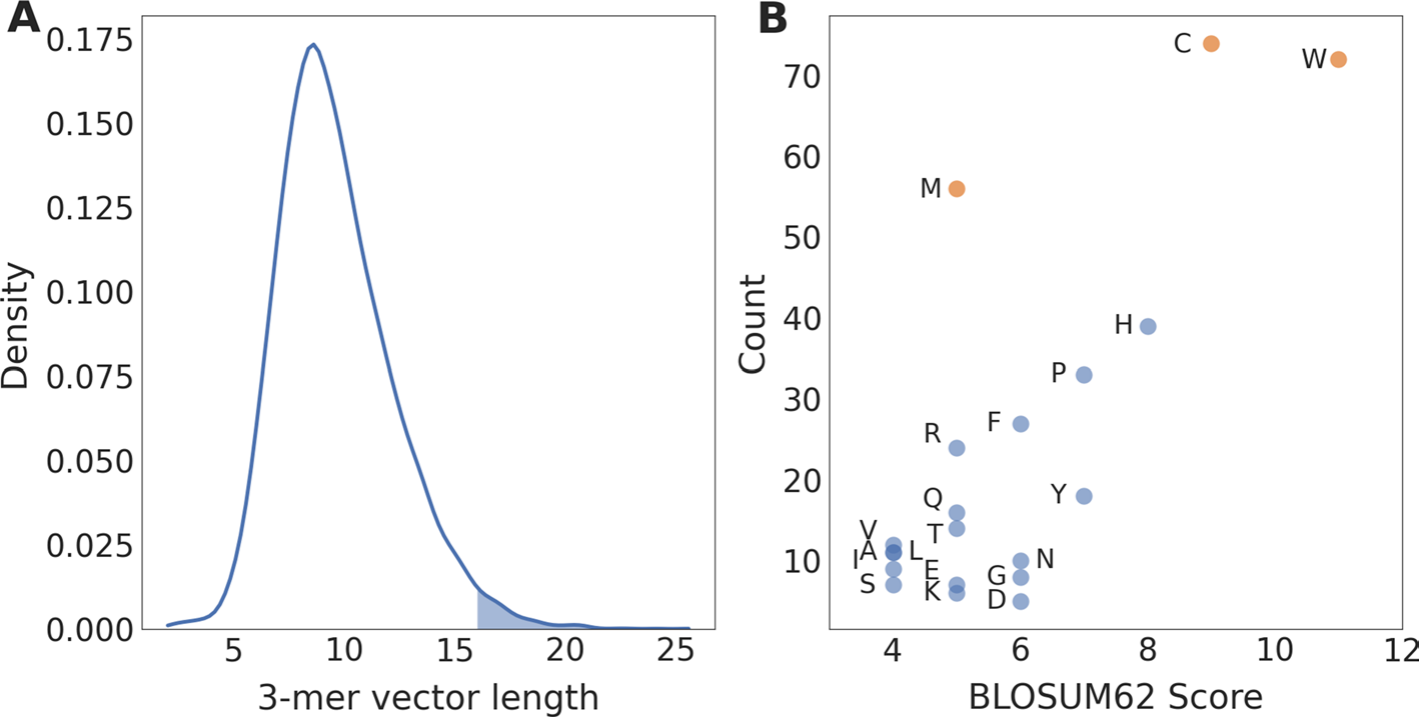
**A** Distribution of the lengths of the 3-mer vectors in the *Bacillus* carbohydrate metabolism Protvec model. The shaded region corresponds to 3-mer vectors with a length greater than 16. **B** Comparison of the *Bacillus* carbohydrate metabolism Protvec model with the BLOSUM62 matrix. The number of occurrences (count) of each amino acid in 3-mer vectors with a length greater than 16 is compared with the value of each amino acid on the diagonal of the BLOSUM62 matrix

First, we investigated the information captured by the learned protein embedding space. Within Protvec models, 3-mer vectors that extend furthest from the origin will have the greatest impact on where sequences are embedded within the protein space. For the Protvec model trained with *Bacillus* carbohydrate metabolism sequences, the distribution of the 3-mer vector lengths, calculated as the Euclidean distance of each vector from the origin, was tailed toward long 3-mer vectors (Fig. 2a). Among the 3-mers within this tail, the amino acids tryptophan (W), cysteine (C), and methionine (M) were most prevalent (Fig. 2b).

### Comparing sequence embeddings

**Fig. 3:**
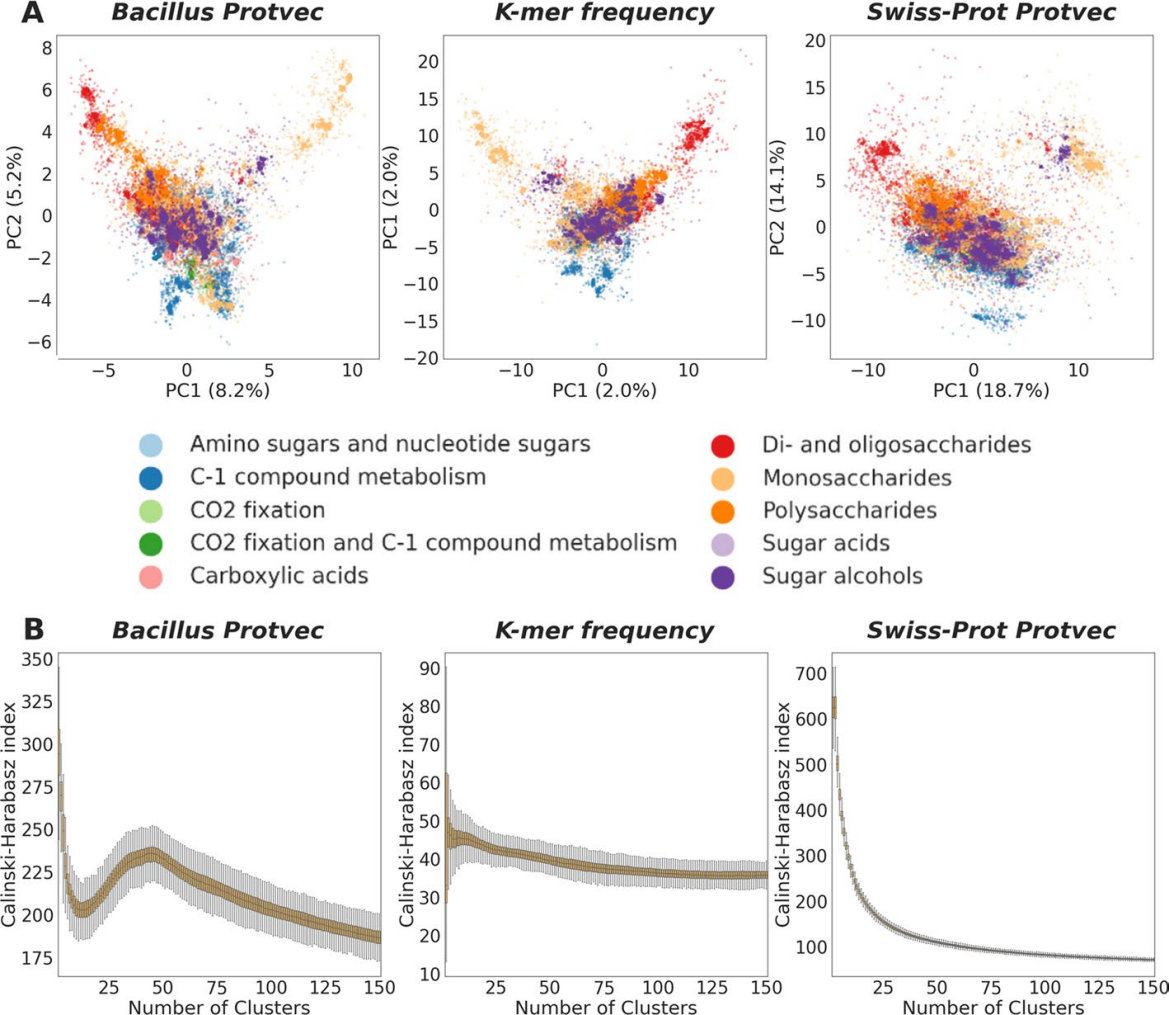
**A** Sequence embeddings of *Bacillus* carbohydrate metabolism sequences embedded using the *Bacillus* carbohydrate metabolism Protvec model, *k*-mer frequency and the Swiss-Prot Protvec model. Sequences are colored by their subclass and visualized using PCA. **B** CH index of *Bacillus* carbohydrate metabolism sequences (n = 5000) embedded using the *Bacillus* carbohydrate metabolism Protvec model, *k-*mer frequency and the Swiss-Prot Protvec model for *K* = 2:150 clusters. For each value of *K*, 500 bootstrap iterations were used

The information used to prepare a sequence embedding may influence how amino acid sequences are grouped within the embedding space and the number of clusters present. To explore how training data influences vector representations of protein sequences, *Bacillus* carbohydrate metabolism sequences were embedded using the *Bacillus* carbohydrate metabolism Protvec model, *k-*mer frequency, and the Swiss-Prot Protvec model (Fig. 3a). Under all three embeddings, sequences belonging to the same SEED subclass were visibly located closely together within the protein space. These clusters appear tightest for the *Bacillus* carbohydrate metabolism model followed by *k*-mer frequency and the Swiss-Prot Protvec model.

The CH index was used to determine whether there is an optimal number of clusters in each embedding where the between cluster variance exceeds the within-cluster variance (Fig. 3b). Using the *Bacillus* carbohydrate metabolism model, the CH index peaks when the embedding is grouped into 48 clusters, however, under the SEED annotation system, these same sequences belong to 29 different subsystems. Furthermore, for the *k*-mer frequency and the Swiss-Prot Protvec embeddings, a point where the between cluster variances exceeds the within-cluster variance is never reached.

### Comparing sequence embeddings with SEED annotations

**Fig. 4:**
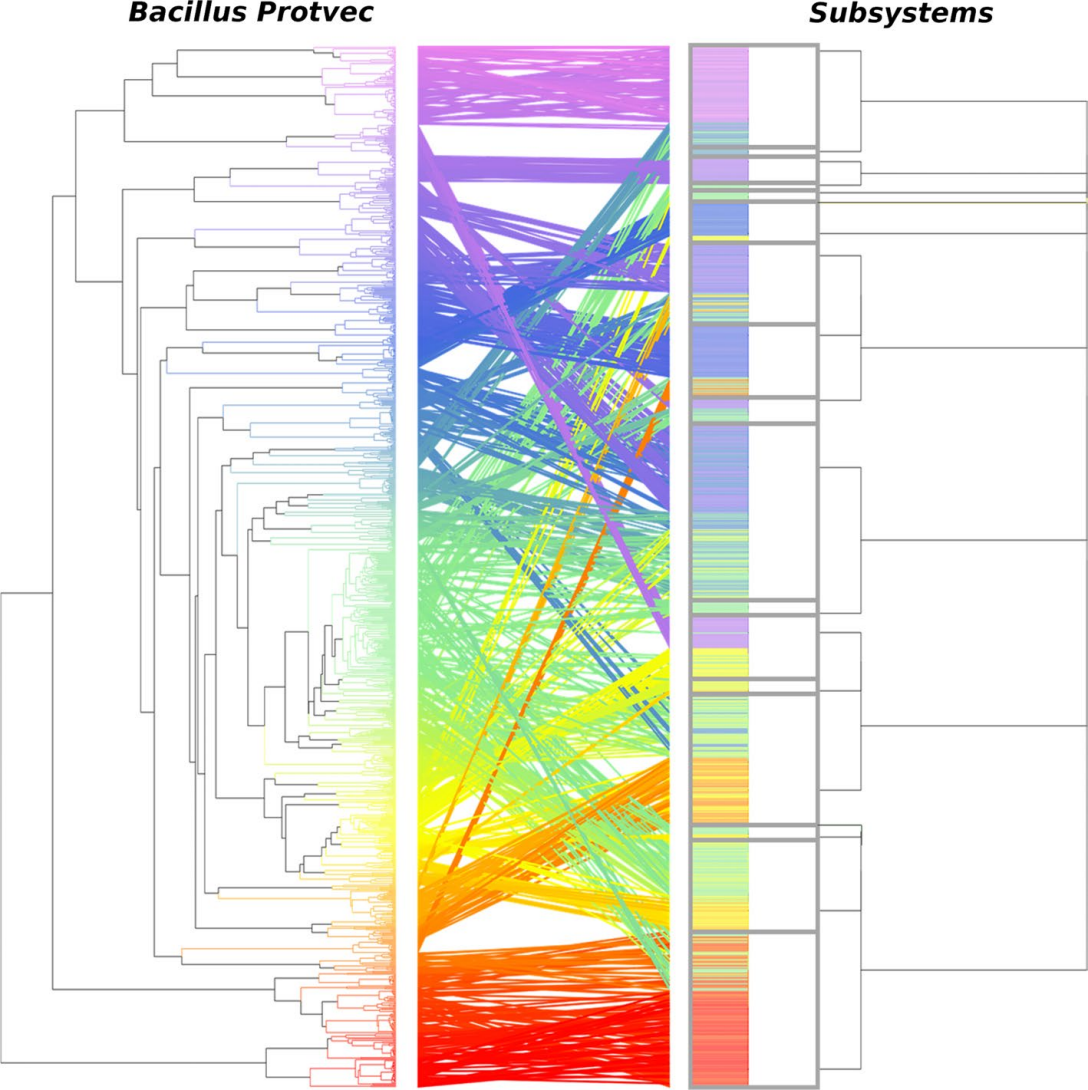
Comparison of *Bacillus* carbohydrate metabolism sequences grouped using agglomerative clustering on sequence embeddings using the *Bacillus* carbohydrate metabolism Protvec model and the SEED annotation hierarchy. The color joining the dendrograms is continuous across the Protvec dendrogram. Boxes are drawn around each subsystem in the SEED annotation hierarchy

Next, we implemented hierarchical clustering to evaluate how the partitioning of sequences using a sequence embedding differed from their SEED annotations. As only the *Bacillus* Protvec sequence embedding demonstrated clear clustering behavior, a hierarchy was built using the Euclidean distance between sequences embedded using the *Bacillus* Protvec model. The tanglegram comparing this hierarchy with the SEED annotation hierarchy shows differences in the organization of sequence functions (Fig. 4). Sequences that were grouped in the embedding-based hierarchy mostly belonged to the same SEED subsystem. However, the higher-level structure was not preserved between hierarchies and closely grouped sequences in the Protvec hierarchy had distant SEED annotations. Additionally, many SEED subsystems consisted of two groups of sequences that belonged to different clades of the embedding-based hierarchy.

### Embedding unknown sequences

As many sequences could not be annotated using SEED functional labels, we embedded *Bacillus* amino acid sequences with unknown functions to cluster sequences with similar but unknown biological roles. As shown previously (Figs. 3 and 4), clustering is improved when a Protvec model specific to the training data is used. Therefore, we trained a Protvec model with unannotated *Bacillus* sequences to embed and subsequently cluster unannotated *Bacillus* sequences.

**Fig. 5:**
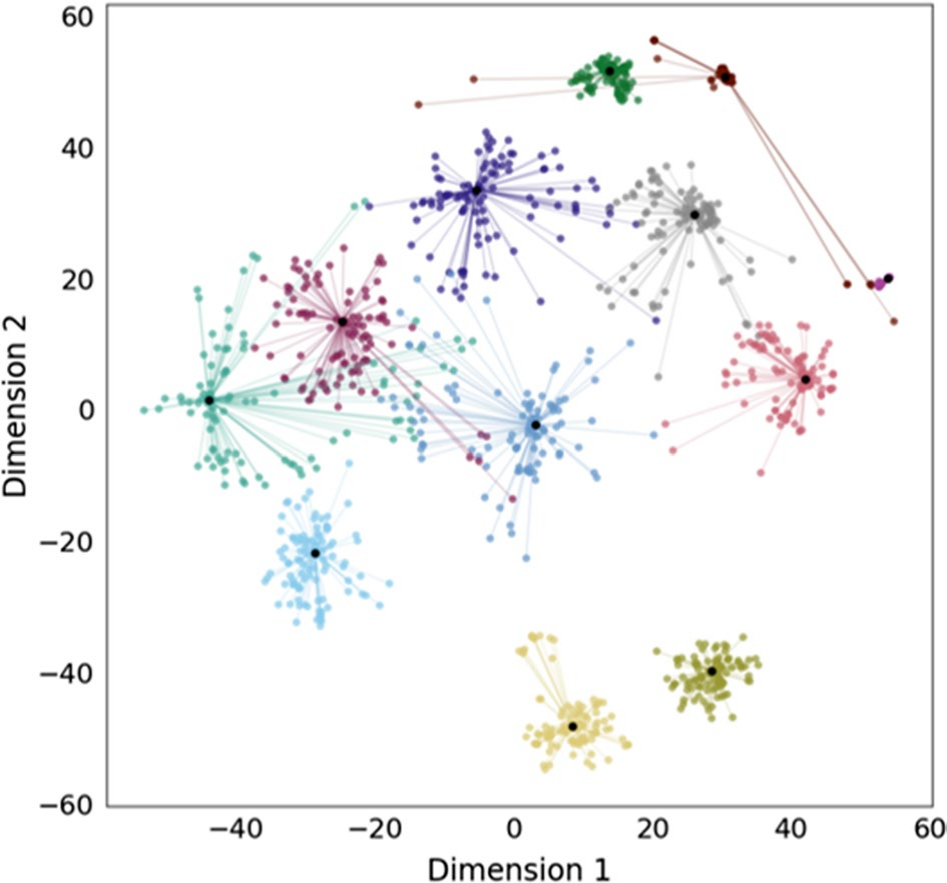
*K*-means clustering of unannotated *Bacillus* sequences embedded using a Protvec model trained with unannotated *Bacillus* sequences. Embedded sequences were grouped into 12 clusters and visualized using *t*-SNE. The 100 sequences closest to the centroid of each cluster are shown in separate colors and the centroid of each cluster is shown in black

Using *k-*means clustering*,* the resulting embedding produced twelve distinct groups of sequences with unknown functions (Fig. 5, Additional File 1: Fig. S2). To verify that these groupings did not arise from homology, the percentage identity between the unknown sequences was calculated (Additional File 1: Fig. S3). The sequence similarity within each unknown cluster was low as demonstrated by the most homologous cluster having a mean percentage identity of less than 50%.

Finally, by embedding *Bacillus* carbohydrate metabolism sequences alongside the unknown *Bacillus* sequences we show that some of these unknown sequences cluster with sequences that have known functions. Despite this, the majority of the unknown sequences group with other unknown sequences (Additional File 1: Fig. S4).

## Discussion

The volume of microbial sequencing data is ever-expanding, with an increasing focus on understanding microbial functions across diverse environments [42]. Despite this, functional annotation ontologies remain incomplete and rely on manual, human-expert curation. In this study, we show using Protvec models, that sequence embeddings can be utilized to evaluate protein annotation ontologies and cluster bacterial protein sequences with unknown functions. Thus, we recommend that sequence embeddings be incorporated into the development of robust annotation schemes for bacterial proteins.

Sequence embeddings are beneficial for understanding functional relationships between proteins as they implement deep unsupervised models which learn protein features, regardless of whether sequence functions are known. These learnt features include the mass, volume, polarity, charge and hydrophobicity of each 3-mer present in a sequence [15]. We further demonstrate the learning capabilities of Protvec by showing that the amino acids tryptophan, cysteine, and methionine have the greatest influence on the location of sequences within the embedding space. Excluding methionine, which plays an important role in protein folding, these amino acids have the greatest similarity scores on the diagonal of the BLOSUM62 matrix used to score the alignment of protein sequences [43]. This indicates that during training, Protvec captures biologically meaningful aspects of amino acid sequences. Similar observations have been made using other sequence embedding techniques including convolutional and long-term short-term memory neural networks which have either mirrored the BLOSUM62 matrix or learned similarities between amino acids with chemically similar sidechains [44, 45]. These learning abilities indicate that sequence embeddings can be generalized across all protein sequences to infer and relate protein functions [21, 46].

While sequence embeddings learn important protein features, these learned features vary for different training datasets and may lead to different vector representations of sequences. We determined that embedding *Bacillus* carbohydrate metabolism sequences with a Protvec model trained with *Bacillus* carbohydrate metabolism sequences resulted in distinct groups of sequences with similar functional annotations. This indicates that using a highly specific, yet relatively small training set (8743 sequences) allowed clustering of functionally similar sequences. However, when these same sequences were embedded using a Protvec model trained with 324,018 sequences from the Swiss-Prot database, which contains functionally diverse sequences across different organisms, clusters of sequences did not form. Other implementations of the *word2vec* algorithm have also determined that word vectors constructed from generalized training data are less effective than domain-specific vector models [47–50]. Therefore, the choice of data used to train sequence embedding models must be carefully considered to produce distinct groups of sequences with shared function. In addition, the sequence clusters formed using the *Bacillus* Protvec model may not have high, between-sequence homology, but rather arise from properties learnt from the training data.

Alternatively, *k*-mer frequency embeddings do not require this consideration as a trained model is not required to embed sequences While *k*-mer profiling produces large matrices which are often memory intensive, we did not detect distinct clusters for the *Bacillus* carbohydrate metabolism sequences embedded using *k*-mer frequency. This demonstrates that Protvec models learn important biological features which are not incorporated into embeddings based only *k*-mer counts. As a result, we suggest that machine learning approaches such as Protvec are used to embed and group amino acid sequences.

Using sequences represented as vectors with Protvec, we show that the sequence groupings produced from the embedding can be used to evaluate a protein ontology. Specifically, we saw that *Bacillus* sequences belonging to the carbohydrate metabolism SEED subclass produced 48 clusters of embedded sequences despite being annotated with 29 different SEED subsystems. Differences between the organization of the embedded sequences and their SEED annotations were further apparent in the hierarchy of the embedded clusters. For example, several SEED subsystems were composed of two separate clusters of embedded sequences and the higher-level organization of the embedded sequences was not consistent with their SEED subclass annotations. These findings indicate that sequence embeddings, based upon algebraic properties of protein sequences differ from functional hierarchies constructed by human experts. Previous work has also shown that clustering sequences using an embedding may produce annotations which contain a higher number of functionally similar sub-groups, allowing more fine-grained annotations to be generated [51]. Consequently, protein sequence embeddings may be used as a framework for designing ontologies that organize sequences mathematically, without needing to rely on experimental observations. While we have shown that sequence embeddings may be beneficial for designing functional ontologies, these improved ontologies will remain incomplete without determining the function of unknown sequences. By embedding *Bacillus* sequences with unknown functions, we identified 12 clusters of proteins with unknown functions. Prime experimental candidates will be selected from each of these clusters and characterized experimentally to infer the function of the remaining sequences in the unknown clusters. As characterizing proteins is expensive and labor-intensive, this represents a more efficient strategy for annotating the entire protein space [52]. Using our approach will greatly expand our knowledge of known protein functions and reduce the growing sequence-function gap by allowing additional labels to be included in bacterial protein ontologies. We also show that some unknown *Bacillus* sequences form clusters with sequences involved in carbohydrate metabolism. This indicates that sequence embeddings may be used as an approach for annotating sequences which cannot be assigned functions using homology alone. In this study, we focus on sequences from *Bacillus* involved in carbohydrate metabolism as a proof of concept. However, future work will use the methods presented to interrogate a broad range of protein functions across diverse microbial taxa to automatically learn new ontologies. Furthermore, since the development of Protvec in 2015, several machine learning methods which represent amino acid sequences as vectors using novel machine learning algorithms have been designed [21–24, 53]. We recommend widespread use of amino acid sequence embeddings to construct protein ontologies which consider various embedding approaches.

## Conclusion

Amino acid sequence embeddings such as Protvec can be used as a systematic framework for developing bacterial protein annotation ontologies. By embedding *Bacillus* proteins involved in carbohydrate metabolism as vectors, we identified inconsistencies between the hierarchical organization of the embedded sequences and their SEED annotations. Furthermore, we grouped protein sequences with unknown biological functions into clusters based on a learnt sequence embedding. These findings indicate that sequence embeddings can be used to design more complete annotation ontologies and develop efficient strategies for discovering unknown bacterial functions.

## Supplementary Information

**Additional file 1** Organizing the bacterial annotation space with amino acid sequence embeddings

## Data Availability

All code employed in this study is publicly available on GitHub (https://github.com/susiegriggo/ProtvecBacterialProteins) including scripts to generate datasets, perform analysis, and generate figures and sequences and output are publicly available on Cloudstor (https://cloudstor.aarnet.edu.au/plus/s/wxRIVLKBejzAutc). Protvec models trained in this study can be accessed at https://doi.org/10.25451/flinders.19770379, https://doi.org/10.25451/flinders.19770742 and the Protvec model trained with sequences from the Swiss-Prot database is available at https://doi.org/10.7910/DVN/JMFHTN.

## Abbreviations

GTDB: Genome taxonomy database
PATRIC: Pathosystems resources integration center
BLOSUM: BLOck SUbstitution Matrix
PCA: Principal component analysis
CH: Calinski-Harabasz
*t*-SNE: *t*-Distributed stochastic neighborhood embedding
