# Supplementary material for: Organizing the bacterial annotation space with amino acid sequence embeddings
Source: BMC Bioinformatics. 2022 Sep 23;23:385. doi: 10.1186/s12859-022-04930-5 (PMC9502642; doi:10.1186/s12859-022-04930-5)
Supplement: Supplementary file 1 — Additional file 1 Organizing the bacterial annotation space with amino acid sequence embeddings [file 12859_2022_4930_MOESM1_ESM.pdf]

Supplementary Material for Organizing the bacterial annotation space with amino acid sequence embeddings

$$\begin{array}{c} \\ FFF \\ FFE \\ FFC \\ \ldots \\ PPP \end{array} \begin{array}{c} \begin{array}{ccccc} FFF & FFE & FFC & \ldots & PPP \end{array} \\ \begin{bmatrix} 1 & 0 & 0 & \cdots & 0 \\ 0 & 1 & 0 & \cdots & 0 \\ 0 & 0 & 1 & \cdots & 0 \\ \vdots & \vdots & \vdots & \ddots & \vdots \\ 0 & 0 & 0 & \cdots & 1 \end{bmatrix} \end{array}$$

Fig. S1 Matrix used to embed amino acid sequences as vectors using *k*-mer frequency.

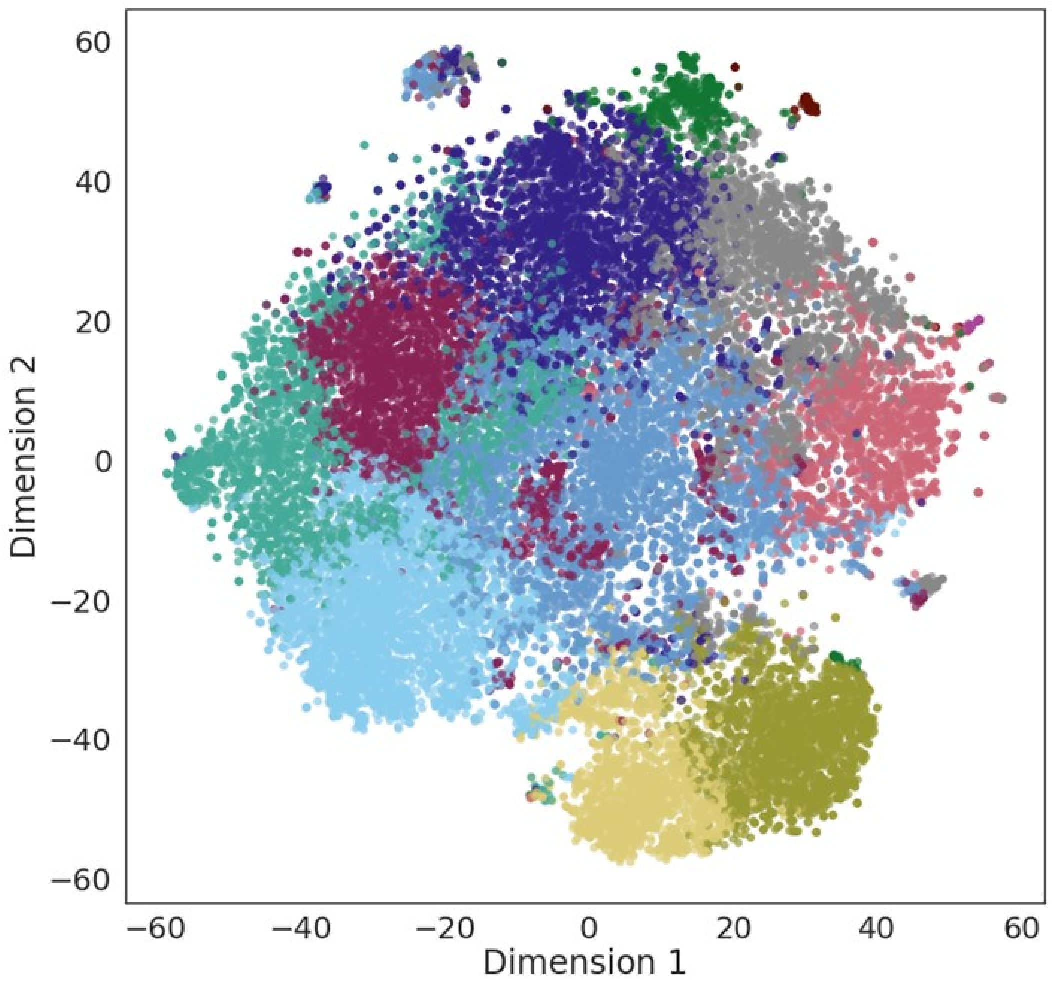

Fig. S2 K-means clustering of unannotated *Bacillus* sequences embedded using a Protvec model trained with unannotated *Bacillus* sequences. Embedded sequences were grouped into 12 clusters and visualized using *t*-SNE.

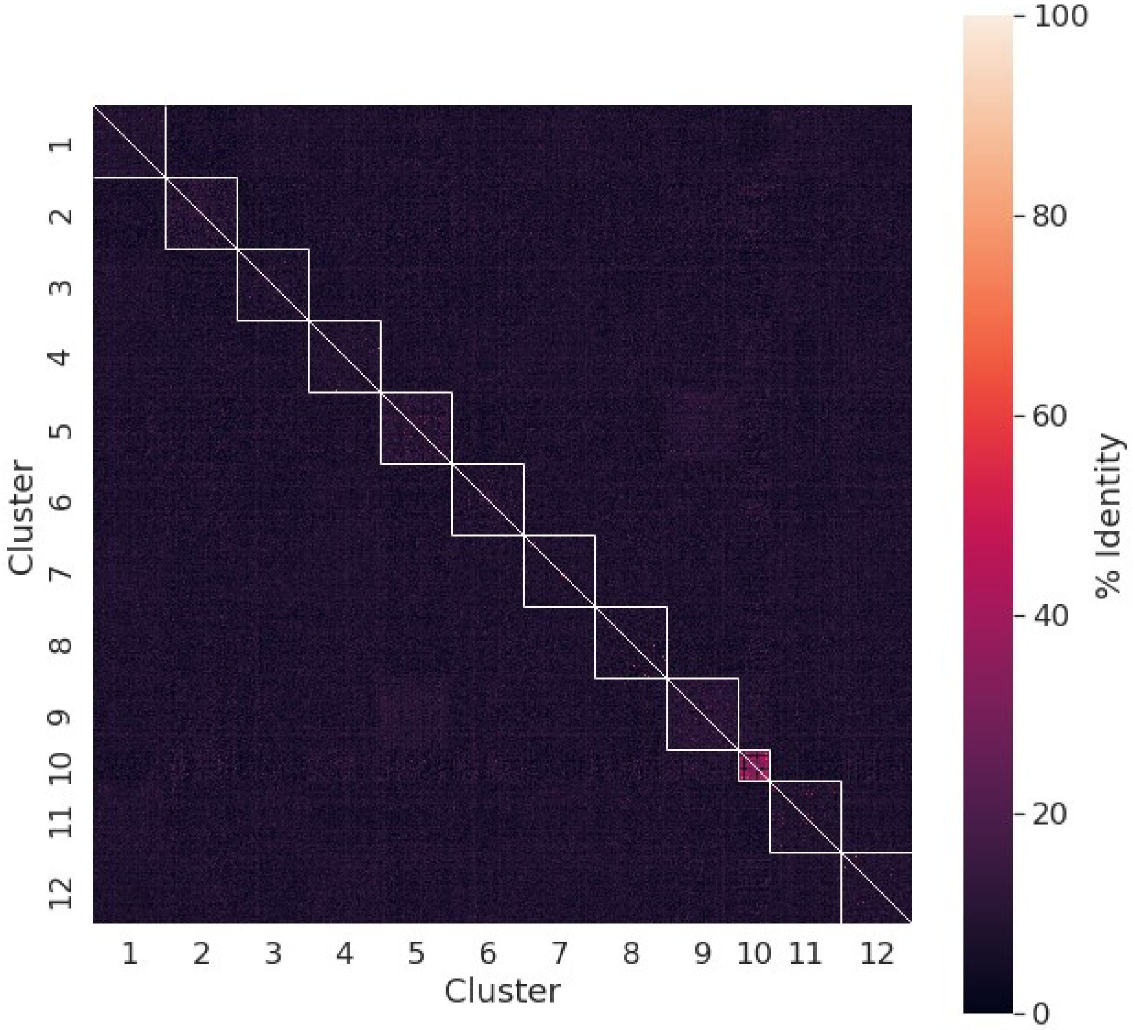

Fig. S3 Per cent sequence identity of unannotated Bacillus sequences clustered using Protvec embedding. For each cluster, the 100 sequences closest to the centroid are shown. White boxes indicate comparisons between sequences belonging to the same cluster.

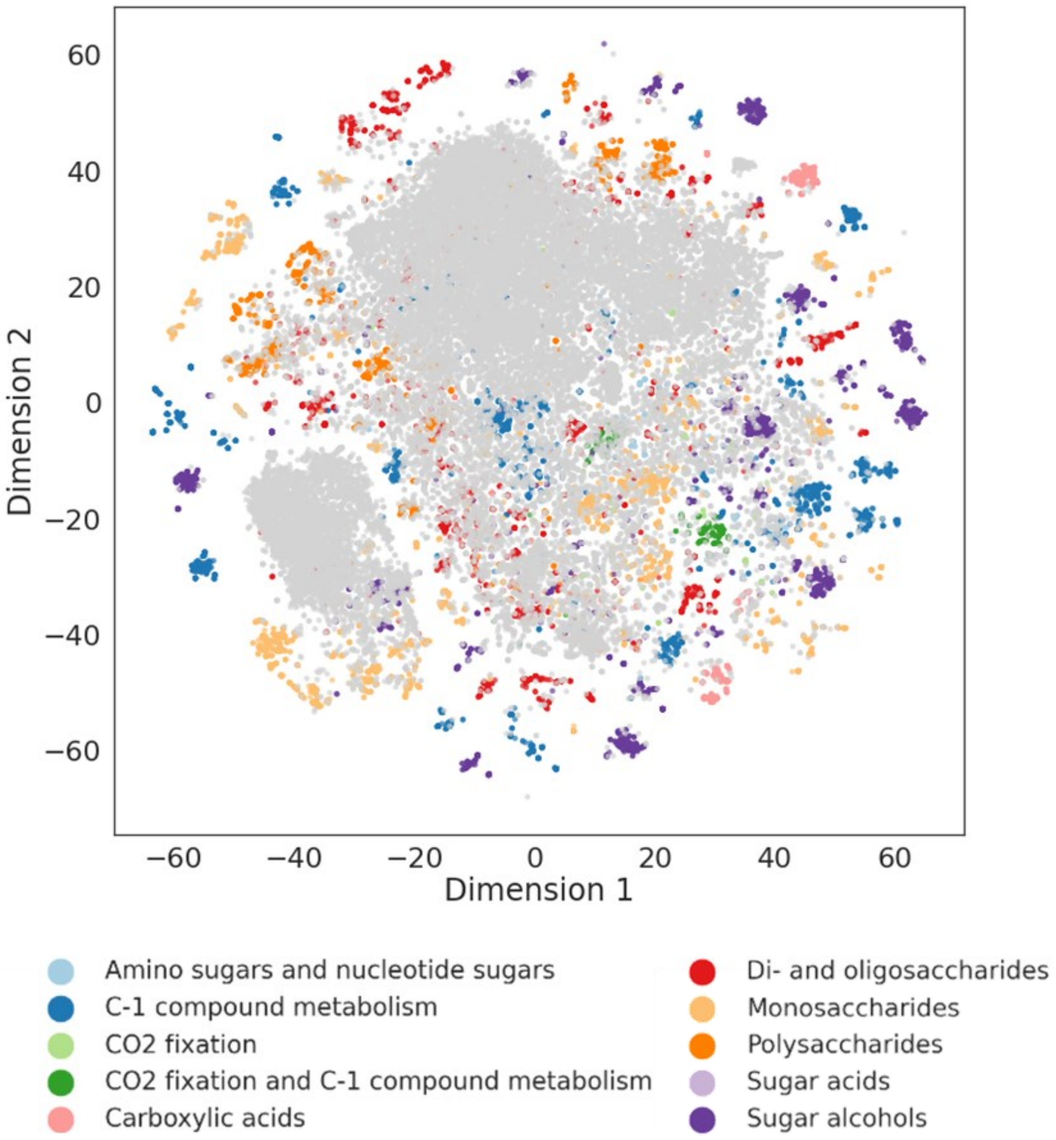

Fig. S4 unannotated *Bacillus* sequences and *Bacillus* carbohydrate metabolism sequences embedded using a Protvec model trained with unannotated *Bacillus* sequences and visualized using *t*-SNE.
